# Cervical Intranodal Schwannoma and Its Malignant Transformation: A Case Report With Literature Review

**DOI:** 10.1002/ccr3.71801

**Published:** 2026-01-04

**Authors:** Shahab Hussain, Zia Ullah Khan, Nazneen Liaqat, Syed Wajihullah Shah, Kainat Khan, Jibran Ikram

**Affiliations:** ^1^ Khyber Teaching Hospital Peshawar Pakistan; ^2^ Department of Medicine Khyber Medical College Peshawar Pakistan; ^3^ Department of Internal Medicine Lady Reading Hospital Peshawar Pakistan; ^4^ Cleveland Clinic Foundation Cleveland Ohio USA

**Keywords:** lymphadenopathy, malignancy, neoplasm, recurrence, schwannoma

## Abstract

This case report highlights a rare malignant transformation of a cervical intranodal schwannoma in a 46‐year‐old man. The patient first came in with a slow‐growing, painless nodule in the right neck. Surgical excision was performed and histopathological examinations revealed a benign intranodal cellular schwannoma. Eighteen months later, the mass recurred and a second surgery showed malignant peripheral nerve sheath tumor (MPNST) at the same site, confirming malignant transformation. This case adds to the limited knowledge of this rare event.

## Key Clinical Message

Cervical intranodal schwannomas, although typically benign, may rarely undergo malignant transformation, presenting as recurrent or enlarging neck masses. Early surgical excision and thorough histopathological evaluation are crucial for diagnosis and monitoring of potential malignant change.

## 1 Introduction

Intranodal schwannomas, also called neurilemmomas, are encapsulated primary mesenchymal tumors that arise within lymph nodes [1]. Although generally benign and non‐metastasizing, rapid growth or associated pain should prompt consideration of possible malignancy [2]. Schwannomas can be found in many anatomical sites, but their primary origin within lymph nodes is extremely rare. Histologically, distinguishing between spindle‐cell lesions in lymph nodes is essential, as it requires careful evaluation due to the potential presence of metastatic disease [3]. Clinically, intranodal schwannomas are often asymptomatic and detected incidentally, typically during histopathological analysis of surgical specimens taken for other indications [4].

We report a patient with a right‐sided neck mass, which was initially diagnosed as an intranodal schwannoma. This rare and exceptional case of malignant transformation highlights the need to consider the possibility of malignancy in schwannomas and emphasizes its importance in the differential diagnosis of atypical nodal masses.

## 2 Case History/Examination

**FIGURE 1:**
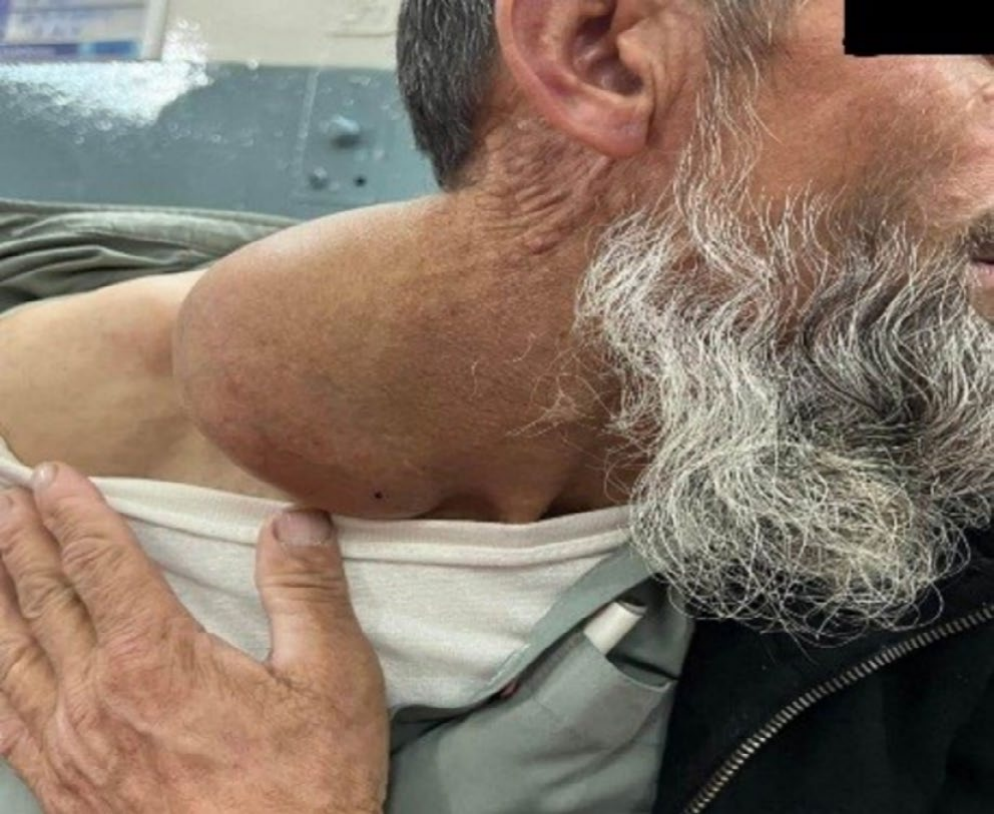
Large, mobile mass on the right side of the neck extending from the clavicle to the mandible.

A 46‐year‐old male presented with a history of a slowly enlarging, painless right‐sided neck mass that had been present for the last 2 years. He also reported multiple subcutaneous swellings on his back and abdomen. Physical examination revealed a large, mobile mass on the right side of his neck, extending from the clavicle to the mandible, with normal overlying skin (Figure 1). MRI showed a complex mass measuring 11 × 8 cm in the right side of the neck, extending into the posterior triangle.

## 3 Differential Diagnosis, Investigations and Treatment

The patient had no clinical stigmata of neurofibromatosis type 1 (NF1), such as café‐au‐lait macules, axillary or inguinal freckling, Lisch nodules, or a family history suggestive of the disorder. Cutaneous and neurological examinations were otherwise unremarkable and no dedicated germline NF1 testing was performed, as the clinical phenotype did not meet diagnostic criteria. Swellings on the back and abdomen were clinically assessed and found to be soft, mobile, subcutaneous lesions consistent with lipomas, with no features suggestive of neurocutaneous syndromes.

**FIGURE 2:**
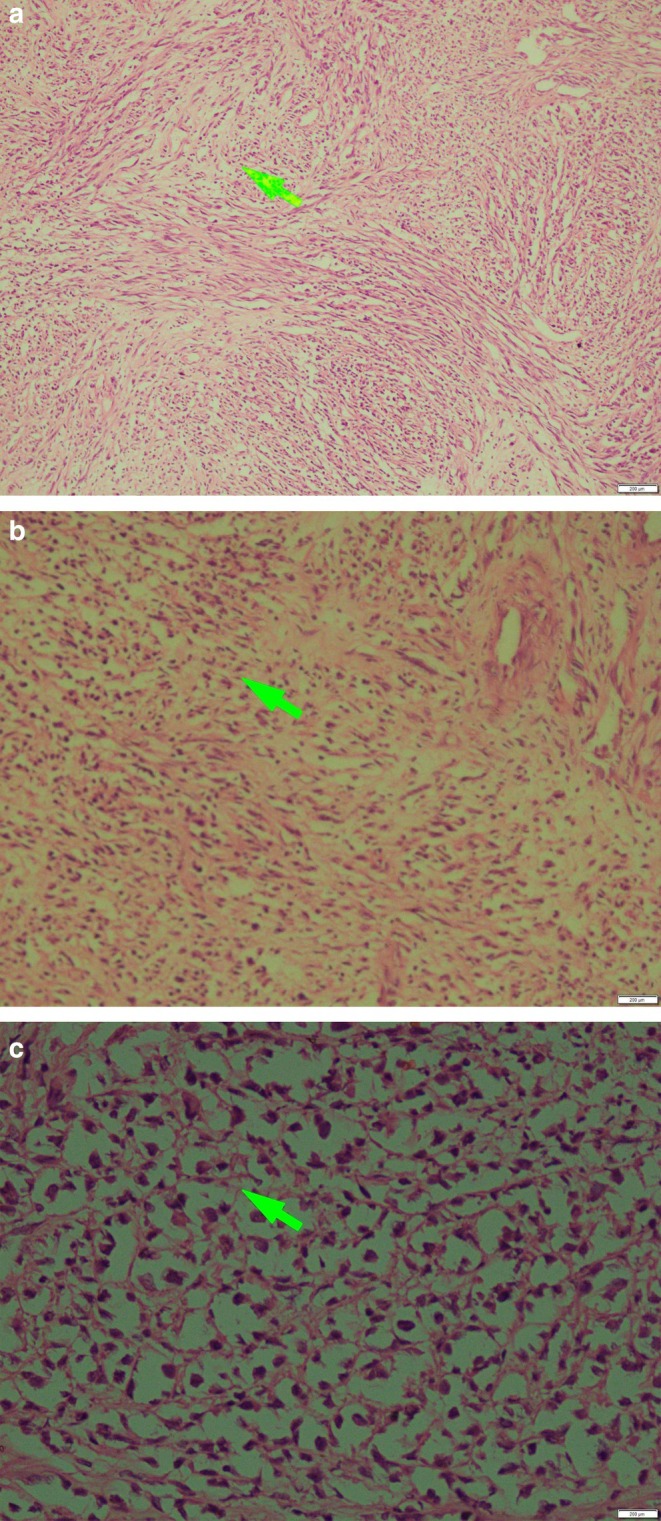
(a) H and E stain (× 100) showing a hypercellular spindle cell tumor with fusiform nuclei arranged in streaming and intersecting fascicles. (b) H and E stain (× 200) demonstrating alternating hypocellular and hypercellular areas with perivascular accentuation; hyperchromatic, plump spindle‐shaped cells with elongated nuclei are arranged in fascicular patterns. (c) H and E stain (× 400) showing a well‐differentiated peripheral nerve sheath tumor with neurofibroma‐like morphology, cytologic atypia and increased mitotic activity.

Surgical excision of the neck mass was performed under general anesthesia but complete removal was not possible due to the mass's adherence and close proximity to vital structures. Gross examination revealed two distinct specimens measuring 148 × 109 × 75 mm and 44 × 32 × 20 mm. The specimens were well‐circumscribed with a gray‐white, lobulated, and fibrotic appearance. Microscopic examination showed the lesion was mainly composed of monomorphic spindle cells with no significant atypia, mitotic activity, or necrosis. Some areas showed varying cellularity, with both hypo‐cellular and hyper‐cellular regions. The spindle cells were arranged in palisading patterns, and the lesion also contained peripheral lymphoid follicles and an infiltrate of lymphocytes. Immunohistochemical staining revealed positivity for S100 and SO × 10, while β‐catenin, Desmin, CD34, and EMA were negative. These findings confirmed the diagnosis of intranodal cellular schwannoma (Figure 2a–c).

## 4 Conclusions and Results (Outcomes and Follow‐Up)

**FIGURE 3:**
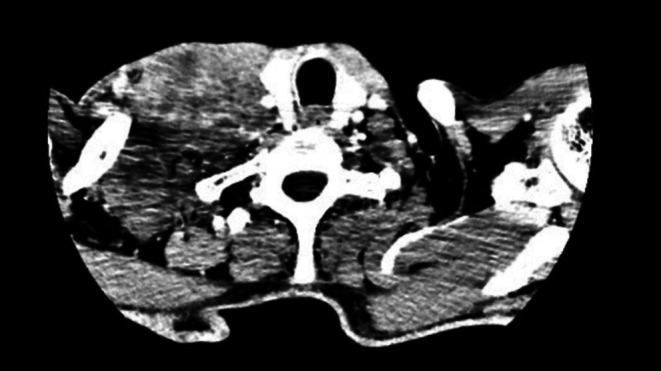
Contrast‐enhanced CT scan of the neck showing a large, ill‐defined lobulated mass in the right supraclavicular fossa and lower neck, abutting the carotid sheath with loss of intervening fat planes and partial encasement of the right subclavian vessels; no adjacent bone erosion is noted.

**FIGURE 4:**
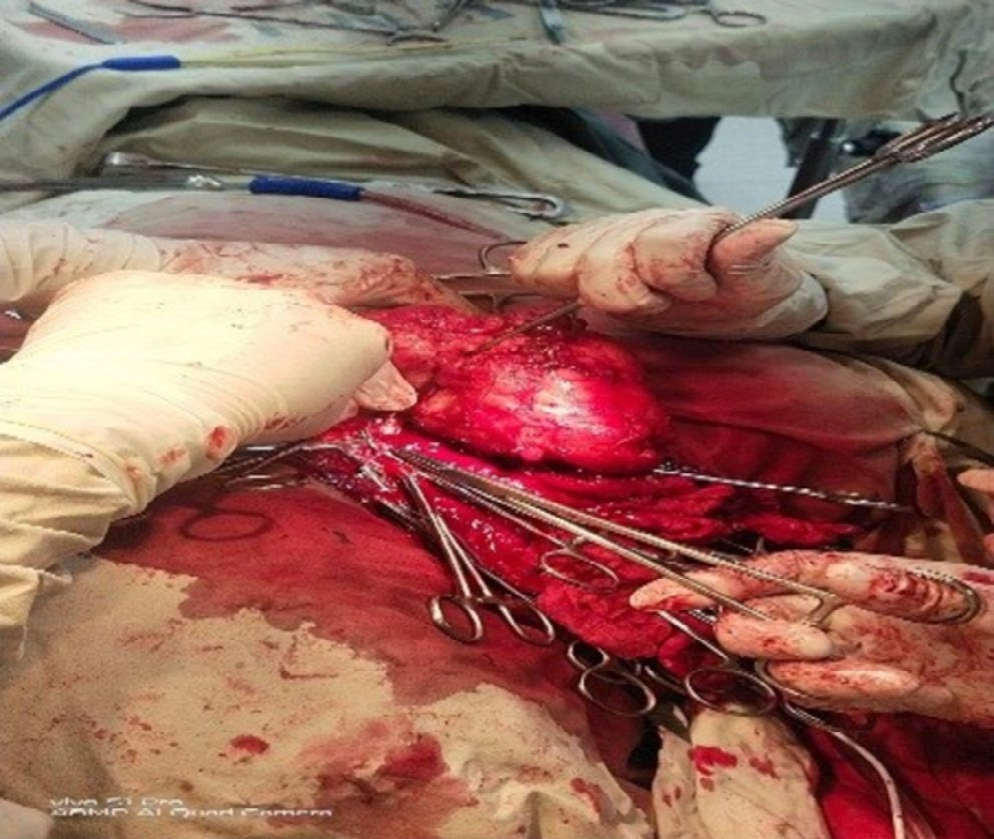
Intraoperative image showing surgical excision of the cervical mass.

Eighteen months later, the patient returned with a right‐sided neck swelling. CT imaging showed a large, ill‐defined lobulated mass in the right supraclavicular fossa and lower neck, measuring 8.4 × 8.8 × 6.9 cm, abutting the carotid sheath and partially encasing the right subclavian vessels. There was no adjacent bone erosion (Figure 3). Surgical intervention was performed again (Figure 4) with maximal debulking achieved. Complete excision was not possible. Gross examination of the specimen revealed multiple tissue fragments, collectively measuring 127 × 117 × 48 mm. Serial slicing of the tissue showed a firm, tan‐white to gray cut surface, with a separate fibro‐fatty tissue piece containing lymph nodes. Microscopic examination revealed a malignant neoplasm with areas of varying cellularity, including regions of hyper‐cellularity and hypo‐cellularity. The tumor cells were spindle‐shaped with hyperchromatic nuclei and arranged in fascicles, with a prominent perivascular accentuation. Foci of myxoid stroma were also present. Immunohistochemical staining showed diffuse, strong positivity for SO × 10. H3K27me3 and INI1 expression was preserved, while CK, ERG, HMB45, and SO × 10 were negative. Biopsy findings confirmed an epithelioid malignant peripheral nerve sheath tumor (MPNST), with two reactive lymph nodes. This diagnosis was consistent with malignant transformation of a pre‐existing intranodal schwannoma, given the patient's previous history of cellular schwannoma.

## 5 Discussion

Schwannomas are benign neoplasms of neurogenic origin that arise from Schwann cells, which are responsible for forming the myelin sheath that insulates neuronal axons [4]. These tumors can develop from any nerve covered by Schwann cells, including cranial nerves (excluding the olfactory and optic nerves), as well as autonomic and peripheral nerves [5]. Schwannomas account for approximately 25%–45% of all tumors found in the head and neck region, with the lateral neck being the most common site of involvement [6]. Grossly, schwannomas are typically encapsulated masses that are eccentrically located relative to the involved nerve. Schwannoma cells are positive for S100 protein, a family of low‐molecular‐weight proteins expressed by neural crest‐derived cells, as well as vimentin, an intermediate filament protein found in mesenchymal cells. These tumors generally lack immunoreactivity for markers associated with smooth muscle or epithelial differentiation [3].

A rare variant of schwannoma is the intranodal schwannoma, which is an encapsulated primary mesenchymal tumor originating within lymph nodes [1]. Intranodal schwannomas typically measure less than 5 cm in diameter, though rare cases exceeding 10 cm have been reported. These tumors usually have a well‐defined, rounded shape and are not adherent to surrounding tissues. Additionally, intranodal schwannomas generally do not present with features such as necrosis, calcification, or cystic degeneration [2]. While these tumors are typically benign, malignant transformation of intranodal schwannomas is extremely rare [5]. Cattani et al. reported the first documented case of a primary malignant schwannoma arising within a lymph node [7].

Recent genomic and molecular studies suggest that malignant progression in peripheral nerve sheath tumors commonly involves disruption of chromatin‐regulatory machinery and tumor‐suppressor pathways rather than a single recurrent oncogene. In particular, inactivation of the Polycomb repressive complex‐2 (PRC2) subunits (e.g., SUZ12 or EED) with resultant loss of H3K27 trimethylation (H3K27me3) has been repeatedly associated with MPNST and with widespread chromosomal instability; concurrent alterations in TP53, CDKN2A and components of the RAS/MAPK and PI3K/AKT/mTOR signaling pathways have also been described, providing a plausible biological route from benign nerve sheath tumors to high‐grade malignancy. These molecular features are important both diagnostically and as potential targets for future therapy [8].

Diagnosing malignant transformation within an intranodal schwannoma can be challenging. Histopathological examination plays a critical role in identifying cellular atypia, increased mitotic activity and necrosis. Immunohistochemical stains for S100 protein and SO × 10 are often used to confirm the schwann cell origin of the tumor [3]. Given the rarity of intranodal schwannomas, a careful differential diagnosis is also essential to distinguish them from other spindle cell tumors. This differential includes metastatic spindle cell tumors, such as sarcomas, spindle cell carcinomas, and melanomas. Among benign intranodal spindle cell lesions, palisaded intranodal myofibroblastoma is a significant consideration [1].

Although intranodal schwannomas are typically isolated, it is important to consider the possibility of associated conditions that may occur with schwannomas. Multiple tumors could indicate neurofibromatosis, and distinguishing between neurofibromas and schwannomas is crucial due to the malignant potential of neurofibromas [9]. Schwannomas are typically encapsulated tumors composed purely of Schwann cells arranged in fascicles and palisades, showing strong S100 and SO × 10 immunoreactivity, as in this case. In contrast, neurofibromas are unencapsulated, composed of mixed cellular elements, and often linked with neurofibromatosis type 1 (NF1), which carries a higher risk of malignant transformation. Immunohistochemistry and careful histological evaluation therefore play a pivotal role in distinguishing these entities [2].

Following clinical suspicion, the diagnosis of schwannomas is usually confirmed through computed tomography (CT) imaging of the affected region, followed by surgical excision and histopathological examination [2]. Definitive diagnosis typically relies on histopathological findings. Surgical resection is the primary treatment for schwannomas due to the risk of local compression and potential malignant transformation [10].

For benign, asymptomatic schwannomas or for patients who are poor surgical candidates, conservative management (observation with serial imaging) or less‐radical procedures may be appropriate; when malignant change is suspected or confirmed, treatment is escalated to oncologic resection and multimodal therapy. Recent head‐and‐neck case series and management algorithms underscore individualized decision‐making that weighs tumor behavior, anatomical constraints, and patient comorbidity [11].

Management of malignant peripheral nerve sheath tumors (MPNSTs) relies on a multidisciplinary, surgery‐first approach. Complete excision with clear margins is the most important determinant of outcome, while adjuvant radiotherapy helps reduce local recurrence in high‐grade or margin‐positive cases. Chemotherapy, typically anthracycline‐ or ifosfamide‐based, is reserved for advanced or metastatic disease and offers limited survival benefit. Emerging options such as MEK inhibitors for NF1‐associated tumors and immunotherapy are under investigation but not yet standard practice. For non‐surgical candidates, palliative radiotherapy or systemic therapy may provide symptom relief [12].

Table 1 summarizes a literature review of intranodal schwannomas, with a focus on the rare occurrence of malignant transformation. The data covers a span of two decades (2002–2022) and includes various patient demographics, tumor characteristics, and clinical presentations.

**TABLE 1:** Summary of intranodal schwannoma cases identified during literature review.

Author	Year	Age	Gender	Site	Size	Duration	Origin	Resection	Malignant transformation
Piana et al. [13]	2002	79y	Female	Lt. mesenteric	40 × 30 × 30 cm	Incidental	Sporadic	Complete	—
Reinus et al. [14]	2004	35y	Male	Lt. adrenal	30 × 40 mm	Incidental	Sporadic	Complete	—
Shayanfar et al. [6]	2008	72y	Female	Perigastric	15 × 15 × 10 mm	Incidental	Sporadic	Complete	—
Grant et al. [15]	2009	67y	Female	Lt. Parotid	42 × 28 × 19 mm	6 months swelling	Sporadic	Complete	—
Ji Han Jung et al. [16]	2009	59y	Male	Retro peritoneum	36 × 32 mm	2 months	Sporadic	Complete	—
Black et al. [5]	2010	77y	Female	Lt. Cervical	15 × 10 × 10 mm	NA	Sporadic	Complete	—
Hayeset al. [17]	2011	13y	Male	Lt. Inguinal	45 × 30 × 25 mm	6–12 months	Sporadic	Complete	—
Rivas et al. [18]	2011	70y	Female	Rt. Hemi thorax	63 × 54 × 52 mm	Incidental	Sporadic	Complete	—
Nam et al. [19]	2011	66y	Female	Lt. Retro peritoneum	100 × 80 mm	NA	Sporadic	Complete	—
Domínguez et al. [20]	2012	42y	Female	Rt. Adrenal	37 mm	Incidental	Sporadic	Complete	—
Nath et al. [1]	2013	42y	Female	Rt. hemi thorax	46 × 36 × 24 mm	18 days dyspnea	Sporadic	Complete	—
Medina‐Gallardo et al. [4]	2017	80y	Female	Rt. mesenteric	7 mm	Incidental	Sporadic	Complete	—
Kang et al. [10]	2019	53y	Female	Rt. Para tracheal	30 × 24 mm	Incidental	Sporadic	Complete	—
Silvestre et al. [3]	2019	69y	Female	Rt. Cervical	57 × 54 × 48 mm	Incidental	Sporadic	Complete	—
Mirea et al. [2]	2022	50y	Female	Rt. Lateral cervical	2 5 × 45 mm	1 year swelling	Sporadic	Complete	—

Abbreviations: cm, centimeters; Lt, left; mm, millimeters; NA, not available; Rt, right; y, years.

Tumor size varied widely, from 7 mm to an impressive 148 × 109 × 75 mm, emphasizing the diverse sizes at which this tumor can occur. The duration of symptoms, when reported, ranged from a few days to several years, indicating the highly variable clinical presentation. In all the cases, the origin of the schwannoma was described as sporadic. All reported cases underwent complete resection, indicating that surgical removal is the standard treatment approach. Importantly, this table underscores the critical issue of malignant transformation in these benign nerve sheath tumors, emphasizing the need to consider this possibility in the differential diagnosis of enlarging or symptomatic schwannomas, particularly in unusual locations such as within lymph nodes.

This case highlights that cervical intranodal schwannomas, though typically benign, may rarely undergo malignant transformation. It underscores the need for complete excision, histopathological vigilance, and long‐term follow‐up to detect recurrence or malignancy early.

## Author Contributions

**Shahab Hussain:** conceptualization, data curation, formal analysis, funding acquisition, investigation. **Zia Ullah Khan:** supervision, validation, visualization. **Nazneen Liaqat:** investigation, methodology, project administration. **Syed Wajihullah Shah:** validation, visualization, writing – original draft. **Kainat Khan:** project administration, resources, software. **Jibran Ikram:** writing – review and editing.

## Funding

No financial support or funding was received for the completion of this case report.

## Ethics Statement

Ethics approval was taken from the relevant institution for the publication of this case report.

## Consent

Written informed consent was obtained from the patient for publication of this case report and any accompanying images. The Wiley standard patient consent form was used.

## Conflicts of Interest

The authors declare no conflicts of interest.

## Data Availability

Data regarding this publication is available upon request from the corresponding author.
